# The Flashed Face Distortion Effect Does Not Depend on Face-Specific Mechanisms

**DOI:** 10.1038/s41598-018-37991-9

**Published:** 2019-02-07

**Authors:** Benjamin Balas, Hannah Pearson

**Affiliations:** 1 Department of Psychology, North Dakota State University, North Dakota, USA; 2 Center for Visual and Cognitive Neuroscience, North Dakota State University, North Dakota, USA

## Abstract

When normal faces are rapidly presented in the visual periphery, they are perceived as grotesque and distorted. This phenomenon, “The flashed-face distortion effect” (FFDE) is a powerful illusion that may reveal important properties of how faces are coded in peripheral vision. Despite the strength of the illusion (and its popularity), there has been almost no follow-up work to examine what governs the strength of the illusion or to develop a clear account of its phenomenology. Presently, our goal was to address this by manipulating aspects of facial appearance and spatial/temporal properties of the flashed-face stimulus to determine what factors modulate the illusion’s strength. In three experiments, we investigated the extent to which local contrast (operationalized by the presence or absence of makeup), image eccentricity, image size, face inversion, and presentation rate of images within the sequence each contributed to the strength of the FFDE. We found that some of these factors (eccentricity and presentation rate) mattered a great deal, while others (makeup, face inversion and image size) made little contribution to the strength of the FFDE. We discuss the implications of these results for a mechanistic account of the FFDE, and suggest several avenues for future research based on this compelling visual illusion.

## Introduction

The “Flashed Face Distortion Effect” (or FFDE) refers to a striking visual illusion in which faces presented sequentially in peripheral vision begin to look increasingly grotesque after just a few faces have been presented^1^. Most observers report large shape distortions, such that faces appear to have strange proportions, as well as distortions of color appearance that lead to faces that look too red, purple, or green. Despite the popularity of the illusion, there is as yet no clear consensus regarding its basis. In the original article describing the illusion, the authors suggest a number of candidate mechanisms that may drive the illusory percept, but to our knowledge there has been very little work to explore the nature of this powerful visual effect. Our main goal, therefore, is to explore the FFDE in more detail, with an emphasis on understanding how the strength of the illusion depends on aspects of face appearance (local contrast of facial features, face inversion) and spatial and temporal factors of stimulus presentation (presentation rate, image size and eccentricity). We continue by discussing the possible relationship between the FFDE and two potential contributing mechanisms: Face adaptation and the limited fidelity of peripheral vision. In each case, we highlight features of these mechanisms that suggest specific manipulations of spatial and temporal parameters that may affect the strength of the FFDE. In the experiments that follow, we implement these manipulations to determine which factors do affect the perceived distortion of faces presented in these sequences, and which factors appear to have a lesser impact.

Because distortion of facial appearance is the key outcome of the FFDE, the literature describing face distortion aftereffects^2^ is a natural place to start trying to understand the mechanisms supporting the phenomenon. Could the FFDE simply be a special case of a face distortion aftereffect (FDAE)? If so, we would expect the strength of the effect to be modulated by spatial and temporal parameters in the same manner as FDAEs. These aftereffects are typically obtained by asking observers to adapt to an image of a face that has been altered to have unrealistic feature proportions (e.g. an elongated nose), warped so that all facial features are compressed or expanded, or had facial features moved within the external contour into unrealistic locations (eyes placed very high in the head, e.g.). After viewing such a stimulus for several seconds or longer, observers typically report that unaltered faces appear distorted in an opposing manner, such that adapting to a compressed face will make a typical face look expanded. These aftereffects have been an important means of studying the underlying nature of population coding for facial appearance^3,4^ and also have yielded insights regarding the category structure of face representation in terms of race, age, and species^5,6^.

There are some challenges to an explanatory account of the FFDE that is based solely on known properties of FDAEs. First, it is unclear from the face adaptation literature whether or not we should expect distortion effects as large as we see in the FFDE following the short presentation times per face that are used in demonstrations of the effect. Usually observers are asked to adapt to a distorted face for at least several seconds before aftereffects are measured, though non-retinotopic figural face aftereffects are measurable in short-duration faces after one second of adaptation^7^. However, the FFDE does not actually incorporate any faces that are truly distorted – instead, the effect must depend on differences in natural appearance between faces in the sequence. It thus seems difficult to adopt this as a primary explanation of the effect. Second, the authors note in the original report that inserting a short blank period between images appears to abolish or at least substantially reduce the effect, which to our knowledge is also not easily related to the adaptation/aftereffect literature. Finally, it also seems problematic that the effect persists (and for some observers appears to increase) as the sequence continues. Observers are capable of estimating the average appearance of a sequence of faces over some interval^8^, and to the extent that prolonged viewing of the sequence is similar to adaptation to the ensemble representation of face appearance, prolonged viewing should lead to adaptation to a face that is fairly average (which is to say, not distorted at all) as the appearance differences between individuals are averaged out. As a result, while distortion aftereffects may be a good place to start in trying to understand the illusion, we also do not think they are a place where we can stop.

The second feature of the FFDE that we chose to consider is the presentation of the images in peripheral vision. Could it be the case that the limited fidelity of peripheral vision plays a key role in the effect? Peripheral vision differs from central vision in a number of ways, and perhaps some of these differences offer a means of understanding the illusory percept, or help address some of the issues facing an account of the effect based on face distortion aftereffects.

We chose to focus on three key differences between peripheral vision and central vision to understand how peripheral viewing may result in the distortions observed in the FFDE. First, visual acuity is substantially poorer in peripheral vision^9^, and both color and contrast sensitivity are worse than in central vision^10^. Second, peripheral vision is also subject to visual crowding, in which the recognition of a target in peripheral vision can be severely impaired by the presence of flanking items within a critical region that scales with target eccentricity^11,12^. Finally, peripheral vision is more sensitive to flicker than central vision, due to the faster responses of rods relative to cones^13^.

Given that the FFDE is not readily observed when the face sequence is viewed centrally, can these characteristics of peripheral vision help us understand why the effect happens? Regarding visual acuity, face images will necessarily look blurrier, etc. as a result of peripheral viewing. It’s not obvious that this should lead to the FFDE, but we also know of no results that make it clear we should rule this possibility out. With regard to visual crowding, the holistic processing of faces makes it difficult to conceive of crowding effects in terms of specific targets and flankers^14^. After all, faces comprising an FFDE sequence are presented in isolation, thus lacking the flanking items that are part of standard crowding tasks. However, recent models of visual crowding that suggest peripheral vision is characterized by a lower-fidelity texture-like representation of visual structure^15–17^ could offer one means by which crowding could contribute to the FFDE. Specifically, in these models of crowding, the hypothesis is that peripheral vision entails a description of stimulus appearance in terms of texture statistics, which only partially constrain the set of possible stimuli that may be present. This ambiguity may lead to some of the distortions we observe in the FFDE as the rapid presentation of each image leaves little time for more than a weak inference regarding the appearance of the stimuli, and the application of ambiguous texture statistics may mean that some distorted faces are equally good candidates for the appearance of each stimulus. Both of these properties of peripheral vision (reduced acuity and increased visual crowding) could therefore contribute to the FFDE, and if they do, manipulating the extent to which images in FFDE sequences are subject to these factors could affect the strength of the effect.

Considering the manner in which face distortion aftereffects and peripheral viewing of FFDE sequences may contribute to the effect leads us to several simple manipulations of the basic FFDE paradigm that have the potential to modulate the strength of the effect. Our goal across the experiments presented here was to implement a set of these manipulations to examine which factors matter and which do not, hopefully leading to a clearer account of why the effect happens in the first place. Specifically, we developed experiments based on the following predictions: (1) If image blur or visual crowding plays a key role in the FFDE, then the size and eccentricity of the faces in peripheral vision should affect the strength of the FFDE. Specifically, increased eccentricity should increase the strength of the illusion and increased size should weaken it. (2) If the temporal properties of face adaptation and aftereffects are an important contributor to the FFDE, than changing the presentation rate of faces within a sequence should also affect the FFDE, and (3) If the reduced contrast sensitivity of peripheral vision is relevant to the FFDE, then manipulating local contrast should also affect the strength of the FFDE. Specifically, increased contrast should weaken the strength of the effect. Finally, (4) If face-specific mechanisms are an important contributor to the FFDE, we would also predict that inverting face images may weaken the effect.

In the experiments that follow, we investigate each of these predictions in turn to better understand the necessary conditions to observe the FFDE. In Experiment 1, we use make-up as a tool for manipulating the local contrast of face images, and examine how this affects the strength of the illusion. We also use inverted face images in this experiment to examine how specific the phenomenon is to upright faces. In Experiment 2, we examine the influence of temporal factors on the FFDE by manipulating the presentation rate of faces within the sequence. Finally, in Experiment 3, we examine how the eccentricity and size of face images in peripheral vision affect the FFDE. Considered together, our results offer novel insights on the nature of the Flashed Face Distortion Effect and also hint at some important avenues for face recognition research based on the striking phenomenology of this illusion.

## Experiment 1

In our first experiment, we investigated the impact of two aspects of facial appearance, local contrast and face orientation, on the strength of the FFDE. Given the reduced contrast sensitivity of peripheral vision, we hypothesized that increasing local contrast in face images might reduce the strength of the illusion. To manipulate the contrast of face images, we chose to present observers with stimuli depicting faces with and without cosmetics. Typical applications of cosmetic products like lipstick, eye liner, and mascara tend to increase the contrast between the eye and mouth regions and the remainder of the face^18^. These contrast relationships are particularly important for a number of face recognition judgments, including attractiveness judgments and sex categorization^19^. Moreover, manipulating local constrast via the presence or absence of makeup has both good ecological validity and also saves us from potential artifacts that arise from globally manipulating image contrast by altering the intensity histogram of a starting image. Besides manipulating contrast, we also hypothesized that if the FFDE depends on face-specific processing, face inversion might also reduce the strength of the effect. Inverting face stimuli tends to reduce performance in a wide range of recognition tasks^20^ and to the extent that the FFDE depends on a contribution from face-specific mechanisms, interfering with those mechanisms by presenting face stimuli upside-down should weaken the distortion effect.

### Methods

#### Participants

We recruited a total of 28 participants (16 female) from the NDSU Undergraduate Psychology Study Pool. All participants were between the ages of 18–24 and self-reported normal or corrected-to-normal vision. None of the participants were familiar with the Flashed Face Distortion Effect prior to participating in the study.

#### Stimuli

To execute our manipulation of local contrast, we chose to use stimuli drawn from the VMU Face Database^21,22^, which is comprised of full-color images drawn from YouTube makeup tutorial videos. We selected 96 pairs of images such that each pair depicted a unique female face without makeup and with makeup applied. The original images were 130 × 150 pixels in size and most images included at least some portion of the external face contour. All images were aligned with respect to the eyes, which may enhance the distortion effect^1^.

#### Procedure

After obtaining informed consent from each participant, the experimenter first explained the nature of the Flashed Face Distortion Effect by showing the participant a version of the illusion that is available on YouTube (http://www.youtube.com/watch?v=wM6IGNhPujE). After confirming that the participant did experience the illusion while watching the video and observed less distortion when the faces were fixated, the experimenter explained that the participant would be shown a series of short image sequences designed to elicit the effect and should rate the strength of the illusion of a 1–7 Likert scale, with “7” indicating very strong distortion and “1” indicating little or no distortion of the faces. Participants were instructed to maintain fixation on a small cross drawn at the center of the display during the task and also told that they could take breaks as necessary by simply withholding their response to the prior stimulus until they were ready to continue.

**Figure 1:**
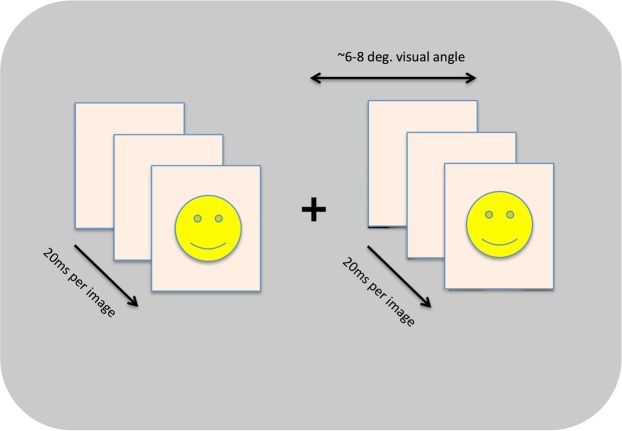
A schematic view of our basic FFDE paradigm. We presented images in rapid succession on either side of a central fixation point, which observers were asked to maintain fixation on during each trial. On each trial, participants were asked to rate the strength of the FFDE on a 1–7 scale after the image sequence concluded. The set of images to be included in the FFDE sequence was randomly sampled on each trial and their order within the sequence was also shuffled randomly on each trial. Across experiments, we varied a range of stimulus parameters within this basic framework including image orientation, the presence/absence of make-up, the duration of images within the sequences, and the eccentricity and size of those images.

All stimulus sequences were presented to participants on a 2560 × 1440 pixel LCD display with a refresh rate of 100 Hz. Participants were seated approximately 40 cm from this display, though viewing distance varied somewhat across participants. On each trial, participants were presented with two sequences of faces presented simultaneously to the left and right of the fixation cross. Each sequence was comprised of a random set of 8 face images drawn on that trial from the larger set of 96 stimuli. These images were each presented 3 times for a total of 24 images in the entire sequence presented on each trial. The order of images within these sequences was shuffled independently for the left and right sequence presented on each trial to ensure that the left and right stimuli were not identical. Each image was displayed for approximately 150 ms with no blank period between consecutive images. Each image was scaled to subtend approximately 4 degrees of visual angle onscreen, and each sequence was presented at an eccentricity of approximately 6–8 degrees of visual angle (Fig. 1). Participants were given unlimited time to rate the perceived distortion of faces in the sequences presented on each trial.

The orientation of the images within sequences (upright or inverted) and the presence or absence of makeup was pseudo-randomized across trials within the design. Participants completed 96 trials per condition for a total of 384 trials in the full testing session. All stimulus presentation and response collection routines were controlled by custom software written using the Psychophysics Toolbox 3 extensions for Matlab^23–25^.

In this experiment (and all that follow), all procedures used in all experiments were approved by the NDSU IRB, in accordance with the guidelines established in the Declaration of Helsinki. Informed consent was obtained from all participants in this and all the following experiments.

### Results

**Figure 2:**
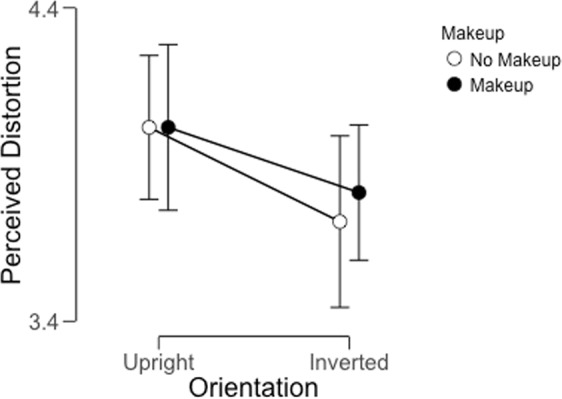
Average distortion ratings across participants for all conditions in Experiment 1. Error bars indicate a 95% credible interval for each data point. While inversion had a weak impact on perceived distortion (see model comparison results in Table 1), makeup had little effect on the strength of the FFDE.

For each participant, we calculated the average rating across all trials in each condition (Fig. 2). We analyzed these values using a Bayesian Repeated-Measures ANOVA implemented in JASP^26^. Table 1 includes the model comparison data obtained from considering models with each main effect included singly, both main effects included, and finally, both main effects and the interaction term included.

**Table 1:** Model comparison output for the results of Experiment 1.

Model Comparison					
Models	P(M)	P(M|data)	BF_M_	BF_10_	error %
Null model (incl. subject)	0.200	0.322	1.903	1.000	
Makeup	0.200	0.066	0.285	0.206	0.932
Orientation	0.200	0.479	3.673	1.485	1.751
Makeup + Orientation	0.200	0.101	0.449	0.313	2.028
Makeup + Orientation + Makeup ∗ Orientation	0.200	0.031	0.130	0.097	10.264

*Note*. All models include subject.

This analysis reveals that there is little evidence in support of a main effect of makeup on perceived distortion. Indeed, the observed Bayes Factor of ~0.20 indicates that there is 5 times more evidence for the null hypothesis than for the alternative hypothesis in this case. With regard to the main effect of orientation, our results are inconclusive: A Bayes Factor of 1.48 indicates weak evidence in favor of the alternative hypothesis (in this case, an effect of orientation on distortion), but this amount of evidence is not typically taken as sufficient to conclude that there is a meaningful effect^27^. Finally, to consider the evidence in support of an interaction between the two factors, we examine the ratio between the Bayes Factor for the full model and the Bayes Factor for the model that includes both main effects^28^. This yields a value of approximately 0.3, which indicates that there is substantial evidence in favor of the null hypothesis in this case.

### Discussion

The results of Experiment 1 suggest that neither the orientation of the faces, nor the presence or absence of makeup impacted the magnitude of the FFDE in this experiment. Regarding makeup, which is our proxy for contrast in this experiment, the results suggest strong support for the null hypothesis (no effect of makeup). We conclude, therefore, that the FFDE probably does not result from the reduced contrast sensitivity of peripheral vision. If this were a key contributor to the FFDE, we would expect that increasing the contrast of our stimuli should have reduced the effect. What about face-specific mechanisms and their contribution to the FFDE? Our results regarding the effect of orientation on perceived distortion are not as conclusive. The Bayes Factor that we obtained for this main effect is in the range of “anecdotal” or “weak” evidence^27^ in favor of the alternative hypothesis (a main effect of orientation on perceived distortion), which means that we cannot draw a strong conclusion regarding either the presence or absence of an effect. We suggest that this means that any effects of inversion on the FFDE are likely rather small, as an inconclusive Bayes Factor often indicates a lack of sufficient power to accept or reject the null hypothesis (no effect of the target manipulation). A more conclusive result may therefore require a larger sample, but presently we conjecture that the potentially small effect size associated with face inversion in this task implies that face-specific mechanisms at least are unlikely to make a substantial contribution to the FFDE, which further suggests that the observed distortion depends on more general properties of spatial vision in the periphery. We continue by examining temporal properties of the FFDE in Experiment 2.

## Experiment 2

In our second experiment, we chose to examine how the duration of images presented within an FFDE sequence impacted the strength of the illusion. Characterizing the effects of image duration on the perceived distortion of faces in FFDE sequences is an important way to examine the effect in the context of known properties of face adaptation (and associated aftereffects), which we suggest is an important candidate mechanism that may contribute to the phenomenon. The strength of face aftereffects depends on both the duration of the adapting stimulus and the duration of the test stimulus. Identity aftereffects grow logarithmically stronger with adaptation duration, and decay exponentially as test duration increases^29^. Distortion aftereffects, which are most relevant to the FFDE, have similar properties^7^. What do these temporal properties imply for the FFDE if adaptation is indeed a contributor to the effect? In terms of face adaptation, a typical FFDE image sequence is somewhat like an ongoing experiment with adaptation and test images being presented in rapid succession, with no interval between them. Increasing the image duration should thus have both positive effects (more distortion) based on the increased duration of each image as an adapting stimulus, but also may have negative effects (less distortion) based on the increased duration of each image as a test stimulus. Despite these opposing effects, we hypothesized that for relatively short image presentation times (on the order of 100–200 ms) the effect of increased adaptation time should be stronger than the effect of increased test image time, leading to more perceived distortion as image duration increases.

### Methods

#### Participants

We recruited a new sample of 34 participants (14 female) from the NDSU Undergraduate Psychology Study Pool. As in Experiment 1, all participants were between the ages of 18–24 and self-reported normal or corrected-to-normal vision. None of the participants were familiar with the Flashed Face Distortion Effect prior to participating in the study.

#### Stimuli

We used the same stimuli as described in Experiment 1.

#### Procedures

All stimulus display parameters and testing conditions were identical to those described in Experiment 1, with the exception of the presentation time for faces within the image sequences presented to participants. In this task, each image within a sequence was either presented for approximately 100 ms, 150 ms, or 240 ms, yielding ‘short,’ ‘medium,’ and ‘long’ sequences respectively, with a total of 64 trials per condition. We also chose to present each participant with either faces with makeup (N = 17) or faces without makeup (N = 17) rather than include this as a within-subjects factor.

### Results

**Figure 3:**
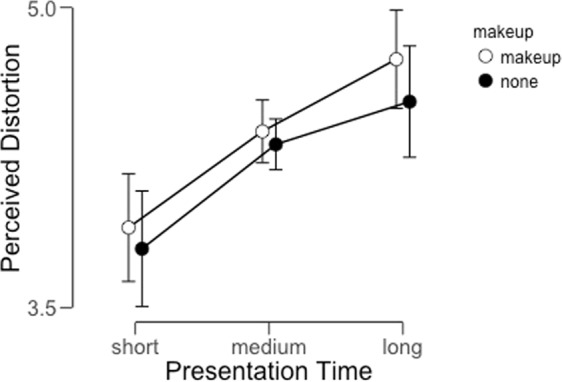
Average perceived distortion across participants for each condition in Experiment 2. Error bars represent 95% credible intervals for each data point.

As in Experiment 1, we calculated the average perceived distortion across conditions for each participant (Fig. 3) and analyzed these values with a Bayesian repeated-measures ANOVA.

This analysis revealed that there was substantial evidence in favor of a main effect of presentation time on perceived distortion (Table 2). The Bayes Factor of approximately 2 × 10^6^ suggests far more evidence in support of the alternative hypothesis (a main effect of presentation time) compared to the null (no effect of presentation time). By contrast, we observed little evidence to support an effect of the presence or absence of makeup on the FFDE. The Bayes Factor in this case was less than 0.5, indicating approximately twice as much evidence in support of the null hypothesis (no effect of makeup on perceived distortion). Finally, to evaluate the evidence in support of an interaction between these factors, we compared the Bayes Factor associated with the full model to the Bayes Factor associated with the model including both main effects, yielding a value of approximately 0.18. This indicates strong support for the null hypothesis (no interaction effect) in this case. Overall, we interpret these results to mean that longer presentation times tend to lead to greater perceived distortion in the FFDE.

**Table 2:** Model comparison outputs for the results of Experiment 2.

Model Comparison					
Models	P(M)	P(M|data)	BF_M_	BF_10_	error %
Null model (incl. subject)	0.200	2.71e −7	1.09e −6	1.000	
Presentation Time	0.200	0.60	6.068	2.221e +6	0.557
makeup	0.200	1.24e −7	4.96e −7	0.456	1.957
Presentation Time + makeup	0.200	0.34	2.03	1.242e +6	2.893
Presentation Time + makeup + Presentation Time ∗ makeup	0.200	0.060	0.26	221806.006	4.254

*Note*. All models include subject.

### Discussion

Our results demonstrate that increased image duration has a straightforward effect on the strength of the FFDE: Within the range of image durations we tested, a longer presentation time leads to more perceived distortion. This is consistent with the temporal dynamics of face adaptation (face distortion aftereffects in particular^7^) which means we cannot reject the possibility that rapid adaptation to images within FFDE sequences induces aftereffects that lead images in the sequence to look distorted. We continue by examining how spatial properties of the images in FFDE sequences (size and eccentricity) may also impact the strength of the effect.

### Experiments 3A and 3B

In our third experiment, we examined how both the eccentricity (Experiment 3A) and the size (Experiment 3B) of the stimuli in FFDE sequences affected the strength of the illusion. We chose to examine these manipulations of FFDE sequences to examine the role of visual crowding and image blur on the strength of the effect. Crowding and blur each limit the information observers can reliably extract from images presented in peripheral vision. In the case of image blur, the way information is limited by peripheral vision is simple: As eccentricity increases, the extent to which high spatial frequencies in the image are available decreases. In the case of visual crowding, the situation is more complicated. When an image is subject to visual crowding, it’s contents tend to appear jumbled or mixed-up^11^, which may be the result of inappropriate averaging of image structure, or the obligatory application of a texture-like code for image appearance that captures statistical information about the image without localizing micro-features of the image precisely^16^. Critically, visual crowding scales with eccentricity subject to Bouma’s Law^12^, such that the image region within which information will be aggregated scales linearly with eccentricity. Varying the size and the eccentricity of images within FFDE sequences thus allows us to manipulate the impact that visual crowding and blur have on the images within each sequence. We hypothesized that if image blur/visual crowding were contributors to the strength of the illusion, increased eccentricity and decreased size should both lead to larger perceived distortion. We examined this in Experiment 3A by keeping the size of the stimuli constant and changing eccentricity across trials, and in Experiment 3B by keeping eccentricity fixed and varying the size of the stimuli presented at that location.

### Methods

#### Participants

We recruited a new sample of 32 participants (12 female) from the NDSU Undergraduate Psychology Study Pool to take part in Experiment 3A and a sample of 56 participants (23 female) to take part in Experiment 3B. As in Experiment 1, all participants were between the ages of 18–24 and self-reported normal or corrected-to-normal vision. None of the participants were familiar with the Flashed Face Distortion Effect prior to participating in the study.

#### Stimuli

We used the same stimuli as described in Experiment 1.

#### Procedures

**Figure 4:**
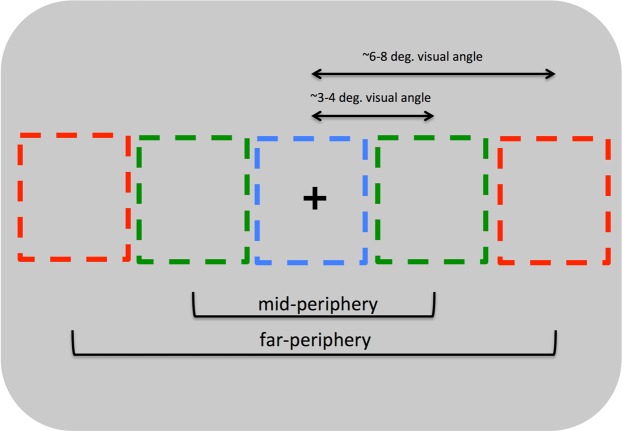
A schematic view of how we manipulated image eccentricity in Experiment 3A. Observers rated FFDE sequences presented either in central vision (blue rectangle), at the eccentricity used on Experiments 1 and 2 (red rectangle), or at an intermediate eccentricity (green rectangle). Image size was not manipulated in this experiment.

Once again, stimulus display parameters and testing procedures were essentially identical to those reported in our first two experiments. In Experiment 3A, however, we introduced variation in eccentricity across trials (Fig. 4) such that participants viewed some sequences centrally, others at the ‘far’ eccentricity we used in Experiments 1 and 2, and still other sequences at a ‘mid’ eccentricity approximately halfway between these locations (3–4 degrees eccentricity).

**Figure 5:**
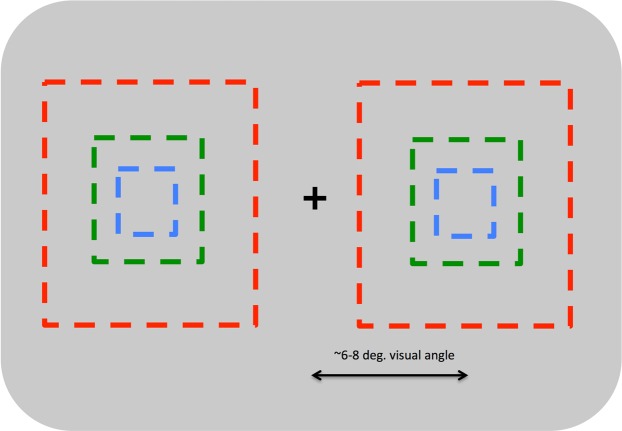
A schematic view of how we manipulated image eccentricity in Experiment 3B. Observers rated FFDE sequences either at the original size (green rectangle), half-size (blue rectangle) or double-size (red rectangle). The eccentricity of the image was fixed at the same distance as Experiments 1 and 2.

In Experiment 3B, all stimuli were presented at the original eccentricity, but the images were either presented at the original size, half of that size, or twice of that size across conditions (Fig. 5). In both experiments, participants completed 64 trials per condition for a total of 192 trials. Also, as in Experiment 2, in both experiments we varied the presence or absence of makeup across participants such that half of the participants in each sample made judgments using faces with makeup and the remainder made judgments using faces without makeup.

### Results

#### Experiment 3A

**Figure 6:**
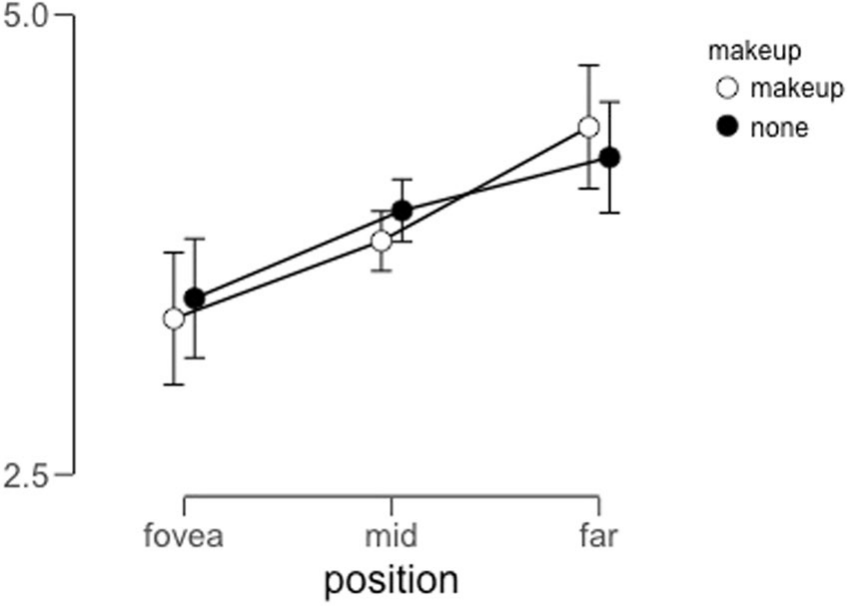
Average perceived distortion within each condition for Experiment 3A. Error bars represent 95% credible intervals for each data point.

We calculated the average rating within each condition per participant (Fig. 6) and analyzed these values via a Bayesian repeated-measures ANOVA.

This analysis revealed strong evidence supporting a main effect of eccentricity on the strength of the FFDE, as evidenced by a Bayes Factor of ~3 × 10^5^ (Table 3). Like we observed in both Experiment 1 and 2, we also found little evidence to support a main effect of makeup (Bayes Factor ~ 0.4, or 2.5 times more evidence favoring the null hypothesis (no effect of makeup)). To examine the evidence supporting an interaction between factors, we calculated the ratio of the Bayes Factor for the full model to the Bayes Factor for the model containing both main effects, and found that this value was approximately 0.28, which indicates about 3.5 times more evidence in support of the null hypothesis (no interaction between makeup presence/absence and eccentricity).

**Table 3:** Model comparison for Experiment 3A.

Model Comparison					
Models	P(M)	P(M|data)	BF_M_	BF_10_	error %
Null model (incl. subject)	0.200	2.281e −6	9.124e −6	1.000	
eccentricity	0.200	0.633	6.885	277303.899	2.569
makeup	0.200	9.005e −7	3.602e −6	0.395	0.859
eccentricity + makeup	0.200	0.288	1.615	126075.238	2.514
eccentricity + makeup + eccentricity ∗ makeup	0.200	0.080	0.347	35024.867	3.551

*Note*. All models include subject.

#### Experiment 3B

**Figure 7:**
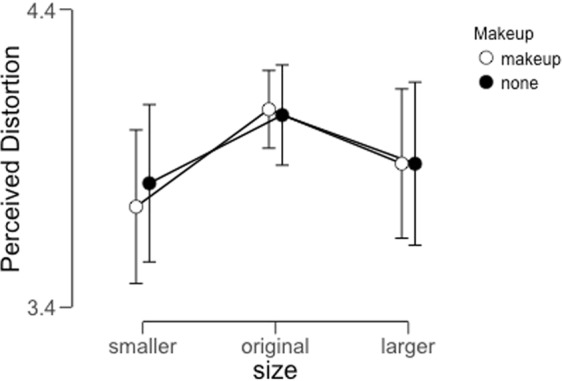
Average perceived distortion within each condition for Experiment 3B. Error bars represent 95% credible intervals for each data point.

The average rating within each condition per participant is displayed in Fig. 7. As in our previous tasks, we applied a Bayesian repeated-measures ANOVA to these values.

This analysis revealed little support for either of the two main effects. The Bayes Factor associated with a main effect of image size was approximately 0.86, which indicates more support for the null hypothesis (no main effect of image size) than the alternative hypothesis (a main effect of image size on perceived distortion). The Bayes factor associated with the main effect of makeup was approximately 0.3, which again indicates strong support for the null hypothesis rather than the alternative hypothesis. Finally, the Bayes Factor associated with the interaction term was approximately 0.09, indicating strong support for the null hypothesis (no interaction between image size and the presence of makeup) (Table 4). We conclude therefore that image size has little effect on the strength of the FFDE. We discuss our interpretation of these results, as well as the results of Experiments 1 and 2, in our General Discussion section.

**Table 4:** Model comparison outputs for the results of Experiment 3B.

Model Comparison					
Models	P(M)	P(M|data)	BF_M_	BF_10_	error %
Null model (incl. subject)	0.200	0.403	2.697	1.000	
size	0.200	0.348	2.132	0.863	0.596
Makeup	0.200	0.124	0.567	0.308	0.515
size + Makeup	0.200	0.113	0.510	0.281	2.694
size + Makeup + size ∗ Makeup	0.200	0.012	0.050	0.031	1.027

*Note*. All models include subject.

## General Discussion

Considered together, our experiments reveal factors that do influence the magnitude of the FFDE and other factors that appear to do little to affect the strength of the illusion. The pattern of results we’ve observed in these tasks suggest important properties of the illusion that provide some insight into the mechanisms that may support the profound distortions of facial appearance that observers see in the illusion. In particular, our goal was to determine what properties of peripheral vision (reduced contrast sensitivity, reduced acuity, visual crowding, etc.) contribute to the effect. We begin by considering the stimulus properties that do not modulate the strength of the illusion.

First, in all of our experiments, the presence or absence of makeup did not have a meaningful impact on the perceived distortion of the faces in our FFDE sequences. For our purposes, makeup is not important in its own right, but provides a useful means of manipulating local contrast within the face via an ecologically valid stimulus. In no case did we observe evidence supporting an effect of makeup on the ratings elicited from our participants. We therefore conclude from this result that the FFDE does not critically depend on the reduced contrast sensitivity of peripheral vision, allowing us to rule out one property of the visual periphery that does not appear to be relevant to the effect.

We also found that inverting the faces presented in the stimulus sequence was of little consequence. This despite the fact that face inversion is well-known to have profound effects on a broad range of face recognition judgments including recognition and discrimination^30^ and even more basic judgments of face shape^31^. These effects are often interpreted as an important demonstration of the specificity of face-sensitive mechanisms: The heavily biased experience in favor of upright faces leads representations of facial appearance to be intolerant of planar rotation, especially in the extreme case of inversion. This also means that face inversion is a useful tool for identifying the contribution of selective mechanisms for face processing. By manipulating face orientation in our first task, we thus hoped to examine the extent to which the FFDE is selective for faces, as opposed to being a reflection of more general properties of visual processing in the periphery. The lack of an effect of inversion in our task (despite evidence that other inversion effects are not limited to central vision^32,33^) suggests that face-specific mechanisms at best make a relatively small contribution to the FFDE (see our discussion following Experiment 1 regarding the interpretation of Bayes Factors between 1–3). We therefore conjecture that the FFDE may depend primarily on general properties of object processing in the visual periphery, or lower-level mechanisms that apply to an even broader class of stimuli. Absent a direct comparison between faces and non-face objects we of course cannot unequivocally conclude that this is the case, but we think this is a reasonable hypothesis given the results we have observed across the experiments reported here. Specifically, we speculate that we should be able to observe a “Flashed Object Distortion” effect with some other class of stimuli, including either non-face objects or abstract shapes that share enough within-class similarity to be comparable to faces in this paradigm. We note that this is largely consistent with previous results describing the dependence of face distortion aftereffects on face orientation: The strength of the aftereffect in these paradigms tends to not be affected by the orientation of the adapting and test stimuli^3,4,34^.

Next, we turn our attention to the stimulus manipulations that did influence the strength of the illusion. First, we found that increasing the amount of time that each image was presented for increased the perceived distortion of faces in the sequence. We suggest that this is consistent with some contribution of shape adaptation mechanisms that may include face-specific populations and more general population codes for complex shapes. The tilt aftereffect, for example, is evident after very short adaptation time^35^ and shape contrast effects are also evident for presentation times on the scale of the FFDE^36^. Further, there is evidence that retinotopic face aftereffects involving radial distortion of face contours are also evident with short adaptation times roughly consistent with our data^37^, though the relevant study involved few participants with some variability across individual observers. Though this offers some explanatory for understanding the FFDE, there are several interesting questions about this presumed link between face and shape aftereffects and the FFDE. In particular, none of these accounts make a clear distinction between central vision and the periphery, so we still have to account for the profound difference in the strength of the illusion with increasing eccentricity. To our knowledge there have not been many studies of how face adaptation and aftereffects work in peripheral vision, but determining the time-dependency of such aftereffects following face adaptation would be an important contribution to our understanding of the basis of this effect.

Regarding the spatial properties of the images comprising FFDE sequences, we observed that the eccentricity (but critically *not* the size of the stimuli) affected the strength of the FFDE. In central vision, participants reported the effect as being quite small, while intermediate eccentricities led to a weaker distortion magnitude than faces presented further out in the periphery. The effect of eccentricity immediately suggests that increased perceived blur may play an important role in determining the strength of the effect. As we mentioned in the introduction, however, visual crowding is another feature of peripheral vision that may also make greater contributions to the illusion with increased eccentricity: Critical regions for crowding scale with eccentricity^11–13^, meaning that more information may be pooled inappropriately within such regions as a stimulus is presented at greater eccentricity. Similarly, scaling the size of the stimulus should also reduce the visual information that is subject to pooling within the Bouma window for visual crowding, ostensibly reducing the distortions associated with low-fidelity encoding of the stimulus. With regard to this latter outcome, our results did not provide much evidence that the manipulation of size had much of an impact, so we suggest that the actual eccentricity of the stimulus (and probably the resulting perceived blur of the face) has more to do with the strength of the illusion than does the extent of visual crowding within a face pattern. This may have to do with specific spatial frequency cut-offs imposed by peripheral vision, though usually we think of face processing as being constrained by spatial frequency defined in terms of cycles across the face^38^. A strong test of the role of blur rather than crowding would be the presentation of blurred images at the fovea in an FFDE sequence, which should lead to a much larger perceived distortion than we observed in the foveal condition of Experiment 3A if blur (rather than crowding) is a key determiner of the effect.

We conclude, therefore, that the Flashed Face Distortion effect is a non-face-specific visual illusion that depends primarily on the increased perceived blur associated with peripheral vision, and also requires sufficient presentation time per image for some amount of rapid mid-level adaptation to face appearance to take place. However, the sheer strength of the illusion still raises several important questions regarding how peripheral object perception differs from what takes place in central vision. As we’ve noted, the temporal scale of the distortion effect seems incongruous with known shape adaptation effects, but perhaps these manifest differently in the periphery. Similarly, it is unclear why prolonged viewing doesn’t ultimately weaken the effect, especially if participants could be adapting to something like the average face across all exemplars included in the sequence. The variety of faces in the sequence does appear to affect the strength of the illusion^39,40^, which suggests some sensitivity to the statistical properties of the sequence, but clearly there remains a good bit about this aspect of the illusion we still don’t understand. Also, while we’ve offered one explanation for the combination of a strong effect of eccentricity with a weak effect of stimulus size, this is also a feature of our data that likely needs more exploration. Overall, we suggest that the Flashed Face Distortion effect is an important platform for future exploration of how faces (and possibly other shapes) are perceived in peripheral vision.

## Data Availability

Data from all three experiments is available via the Open Science Framework.
